# A Nucleotide Metabolism-Related Gene Signature for Risk Stratification and Prognosis Prediction in Hepatocellular Carcinoma Based on an Integrated Transcriptomics and Metabolomics Approach

**DOI:** 10.3390/metabo13111116

**Published:** 2023-10-30

**Authors:** Tianfu Wei, Jifeng Liu, Shurong Ma, Mimi Wang, Qihang Yuan, Anliang Huang, Zeming Wu, Dong Shang, Peiyuan Yin

**Affiliations:** 1 Clinical Laboratory of Integrative Medicine, First Affiliated Hospital of Dalian Medical University, Dalian 116000, China; tianfuwei0901@gmail.com (T.W.);; 2 Department of General Surgery, First Affiliated Hospital of Dalian Medical University, Dalian 116000, China; 3 Institute of Integrative Medicine, Dalian Medical University, Dalian 116000, China; 4 iPhenome Biotechnology (Yun Pu Kang) Inc., Dalian 116000, China

**Keywords:** nucleotide metabolism, hepatocellular carcinoma, prognosis signature, molecular classification, chemotherapy sensitivity, tumor immune microenvironment

## Abstract

Hepatocellular carcinoma (HCC) is a leading cause of cancer-related mortality worldwide. The in-depth study of genes and metabolites related to nucleotide metabolism will provide new ideas for predicting the prognosis of HCC patients. This study integrated the transcriptome data of different cancer types to explore the characteristics and significance of nucleotide metabolism-related genes (NMGRs) in different cancer types. Then, we constructed a new HCC classifier and prognosis model based on HCC samples from TCGA and GEO, and detected the gene expression level in the model through molecular biology experiments. Finally, nucleotide metabolism-related products in serum of HCC patients were examined using untargeted metabolomics. A total of 97 NMRGs were obtained based on bioinformatics techniques. In addition, a clinical model that could accurately predict the prognostic outcome of HCC was constructed, which contained 11 NMRGs. The results of PCR experiments showed that the expression levels of these genes were basically consistent with the predicted trends. Meanwhile, the results of untargeted metabolomics also proved that there was a significant nucleotide metabolism disorder in the development of HCC. Our results provide a promising insight into nucleotide metabolism in HCC, as well as a tailored prognostic and chemotherapy sensitivity prediction tool for patients.

## 1. Introduction

Hepatocellular carcinoma (HCC) is one of the dominant types of cancer all over the world [1]. HCC is the third leading cause of mortalities among all malignancies in the world [2,3,4]. In addition, effective prognostic indicators would be a boon for these patients. Thus, it is urgent to develop and verify new prognostic signals to predict the clinical prognosis of HCC patients at an early stage in order to improve the survival rate of patients.

Nucleotide is the basic building block of organisms, and it is an essential raw material for producing nucleic acid to sustain cell proliferation [5]. Nucleotide metabolism is in a state of dynamic equilibrium, which is important for maintaining normal physiological functions of cells [6,7]. Recently, researchers have affirmed that abnormal nucleotide metabolism enhances the growth of tumors and suppresses the normal immune responses in the tumor microenvironment [8]. For example, disrupting the homeostasis of the pools of nucleotides can produce mutations that influence antigen presentation and, ultimately, the immune response to the tumor [9,10]. Targeting nucleotide metabolism also provides new directions for the development of novel antitumor-specific drugs [11,12]. Therefore, focusing on the reprogramming of the nucleotide metabolism will provide new ideas for predicting prognostic outcomes in HCC patients. Moreover, the clinical relevance of nucleotide metabolism-related genes (NMRGs) in predicting outcomes and guiding chemotherapeutic strategies for patients with HCC remains unknown to the best of our knowledge. Thus, the development of the HCC risk stratification tool using NMRGs is promising.

In the present research, we will systematically evaluate the potential of NMRGs in predicting the prognosis of HCC patients using a bioinformatics approach and establish a risk score signal based on NMRGs to predict the clinical outcome of HCC patients. This model could be utilized in making clinical decisions and providing individualized care. To further validate the credibility of the model, we examined the expression of NMRGs in the model at the cellular level by molecular biology experiments. Ultimately, we used non-targeted metabolomics to detect the nucleotide metabolism-related products in serum samples of patients with HCC, further supporting our study from the metabolic point of view. We are optimistic that the findings of this investigation will avail a greater and new insight into the diagnosis and management of HCC. Additionally, it will be essential in availing a theoretical basis for upcoming nucleotide metabolism studies.

## 2. Materials and Methods

### 2.1. Data Collection and Processing

Firstly, 97 NMRGs were obtained based on the following dataset from the Molecular Signatures Database (MSigDB): REACTOME_METABOLISM_OF_NUCLEOTIDES. RNA-sequencing (RNA-seq) and the matched clinical characteristics were derived from the TCGA database. The samples that were obtained contained 373 and 49 HCC patient samples and normal samples, respectively. RNA-seq, along with clinical data obtained from the Gene Expression Omnibus (GEO) database (GSE14520), were used for external validation. Patients who did not have information on their survival were excluded from further analysis. To facilitate batch normalization, the “sva” package in R was employed. In addition, the TCGA database was another database that was utilized to acquire SNV, transcriptome profiles, CNV, methylation data, and pan-cancer transcriptomes’ clinical features.

### 2.2. Pan-Cancer Analysis

Currently, inadequate research has been conducted to determine the link between nucleotide metabolism and malignancies. As a result, the differences in NMRGs in various malignancies are described inadequately. SNV, CNV, methylation, and mRNA expression data were examined and graphically illustrated as heatmaps to avail a pan-cancer summary of NMRGs. Moreover, a univariate Cox regression analysis between the mRNA expression and OS to probe into the value of NMRGs in the prognoses of patients with various malignancies was conducted using R version 4.0.3 and TBtools version 1.098 [13].

Single sample gene set enrichment analysis (ssGSEA) was used to calculate NMRG scores in every sample of each cancer to reveal the differential function of pathways regulated by NMRGs in various kinds of human tumors. Samples were categorized into two groups, one with the top 30% of NMRG scores and the other with the worst 30%. Gene set enrichment analysis (GSEA) was used to investigate the differences in pathway activity between the two groups based on the transcriptomes of the two groups.

### 2.3. Differentially Expressed Prognostic NMRG Identification

The “limma” packages were utilized to uncover the differentially expressed NMRGs between HCC and normal tissues (FDR < 0.05, fold change > 1.5). Next, 97 NMRGs screened out were put into univariate Cox regression analysis to acquire the genes with prognostic significance (*p* < 0.05). Afterward, the intersection of the two sets of genes was taken to obtain 32 NMRGs for subsequent analysis, as shown by the Venn diagram.

### 2.4. Non-Negative Matrix Factorization (NMF) Clustering Determination of NMRG Modification Subtypes

The HCC samples from the TCGA database were clustered by the NMF based on the expression data of 32 NMRGs. The range for the cluster count, k, was set from 2 to 10. The R package “NMF” calculated the common membership matrix’s average contour width. On the basis of the dispersion, cophenetic, and silhouette metrics, the ideal cluster numbers were established. Afterward, the samples are split into two distinct molecular subtypes C1 and C2.

### 2.5. Gene Set Variation Analysis (GSVA) and NMRGs Different Expression Analysis

The NMRG scores of individual patients with HCC were computed by the “GSVA” package in R, which could serve as an indicator of nucleotide metabolism activities. Then, the “Wilcox.test” function in R and a T-test were employed to compare the difference in the scores and expression of NMRG between two clusters, respectively.

### 2.6. Differences in the Prognosis, Immune Checkpoint Genes, and Drug Sensitivity between Distinct NMRG-Based Clusters

The prognostic efficacy of clusters was assessed using Kaplan–Meier analyses, with the progression-free interval (PFI), disease-specific survival (DSS), disease-free interval (DFI), and overall survival (OS), as standards. Subsequently, the “Wilcox.test” function in R was adopted to explore the disparity between infiltration levels of typically immune checkpoint genes (ICGs). Additionally, we used pRRophetic [14], the R software that predicts the clinical chemotherapeutic response utilizing the expression levels of tumor genes, to calculate the semimaximum inhibitory concentration (IC50) of commonly used chemotherapeutic drugs in the HCC cohort. A Wilcoxon signed-rank test, on the other hand, determined if the difference in the IC50 between two clusters is statistically significant. A decreased semi-inhibitory mass concentration of the drug in malignant cells is always associated with a smaller IC50, indicating that the cancer cells are more susceptible to the medicine.

### 2.7. DEG Identification and Functional Analysis

DEGs between two clusters based on NMRGs were identified by the limma package, with the thresholds established as FDR < 0.05 and fold change > 1.5, which was further subjected to the Kyoto Encyclopedia of Genes and Genomes (KEGG) pathway and GO functional enrichment analyses using the R package “clusterProfiler”.

### 2.8. Construction and Verification of a Prognostic Signature Based on NMRGs

The 32 differentially expressed and prognostically significant NMRGs obtained previously were incorporated in the least absolute shrinkage and selection operator (LASSO) Cox regression signature to develop the powerful prognostic signature. The risk score of each patient is calculated using the “Prediction” function in R, and then HCC patients in TCGA and GEO groups were classified into high- and low-risk groups as per the median risk score, and comparisons of their prognoses were done. To additionally test the viability of the risk score-based predictive signature in patients with HCC in the TCGA as well as GEO datasets, the principal component analysis (PCA) and the t-distributed stochastic neighbor embedding (t-SNE) analyses were done. Using the “survival ROC” R package version 4.0.3, time-dependent receiver operating characteristic (ROC) curves and AUC values were obtained to ascertain the specificity and sensitivity of the risk score.

### 2.9. Creating a Predictive Nomogram That Incorporates Clinical Characteristics and Risk Scores

The clinical data, which comprised age, gender, grade, and stage as well as the risk score of every patient in TCGA cohorts, were retrieved. The statistically significant indicators (*p* < 0.05) from the univariate Cox survival analysis of each indicator were then incorporated into the multivariate Cox survival analysis. These markers were regarded as independent prognostic variables (*p* < 0.05) in the multivariate Cox survival analysis. A nomogram was constructed utilizing the above clinical features and risk score. The nomogram’s discriminating power and prediction accuracy were then assessed using calibration curves. The prediction performance was also assessed using the time-dependent ROC curve.

### 2.10. Reagents

Cell culture-related reagents such as Dulbecco’s Modified Eagle Medium (DMEM), Minimum Essential Medium (MEM), and Roswell Park Memorial Institute 1640 Medium (RPMI-1640) were purchased from Gibco (Grand Island, NE, USA). PCR-related reagents were purchased from Accurate Biology (Changsha, China). Methanol, isopropanol, acetonitrile, formic acid, and ammonium acetate of mass spectrometry grade were supplied by Fisher Scientific (Fair Lawn, NJ, USA). Ammonium bicarbonate and methyl tert-butyl ether (MTBE) of mass spectrometry level were purchased from Sigma-Aldrich (St. Louis, MO, USA). Ultra-pure water (18.2 MΩ) was prepared by a Milli-Q water purification system (Merck KGaA, Darmstadt, Germany).

### 2.11. Cell Culture

The human HCC cell lines (HuH7, HepG2, and Hep3B2.1–7) were purchased from Procell Life Science & Technology (Wuhan, China). The L-02 cell line (human normal hepatocytes) was purchased from BeNa Culture Collection (Beijing, China). Briefly, HuH7 and L-02 were, respectively, grown in DMEM high glucose medium (Gibco, Grand Island, NE, USA) and RPMI-1640 medium, while HepG2 and Hep3B2.1–7 were incubated in MEM medium, all of which containing 10% fetal bovine serum and 1% penicillin-streptomycin solution. All the cells were incubated in a cell incubator under 37 °C with a concentration of 5% CO_2_.

### 2.12. Real-Time Quantitative Polymerase Chain Reaction (qPCR)

The total RNA in the HuH7, HepG2, Hep3B2.1–7, and L-02 cell lines was extracted by the conventional Trizol method and the cDNA was obtained using a reverse transcription kit (Accurate Biology, Changsha, China). Furthermore, the expressed level of target gene was detected by using SYBR Green I fluorescent dye-based assay and β-actin was used as the internal reference gene. RNA level was analyzed and quantified by 2-ΔΔCt. The primer sequences of the genes were shown in Supplementary Table S1.

### 2.13. Participants and Criteria

Serum samples from HCC patients (*n* = 26) and healthy individuals (*n* = 26) were obtained from the biological sample bank of the First Affiliated Hospital of Dalian Medical University (collected from November 2016 to December 2019). In addition, the study has been approved by the Ethics Committee of the First Affiliated Hospital of Dalian Medical University (No. PJ-KS-KY-2021–129). Inclusion criteria for the HCC group included: (1) signed informed consent for collection and use of biological samples and aged ≥18 years; (2) the pathological diagnosis is HCC; (3) follow-up information is complete; (4) no other malignant tumors and no prior anti-tumor treatment was performed before surgery; (5) the biological sample is complete. Exclusion criteria include: (1) new adjuvant or chemical therapy before surgery; (2) accidental death during operation or postoperative relapse resulting in death within one month; (3) the follow-up information is incomplete or the biological sample is missing. Serum samples from the control group (CON group) were obtained from healthy individuals on physical examination and matched the sex and age composition of the HCC group.

### 2.14. Serum Sample Pretreatment and Non-Targeted Metabolomics Analysis

The pretreatment procedures of serum samples were divided into two parts, namely, extraction of polar small molecule metabolites and lipids. Briefly, to extract the polar metabolites, we added 150 μL of the serum sample to a 96-DeepWell plate followed by 600 μL of methanol solution. After the mixture was vortexed for 5 min, it was centrifuged at 5300 rpm for 20 min. The supernatants were divided into two 200 μL aliquots, and transferred to two individual 450 μL 96-well plates, and the liquid was lyophilized by a freeze dryer. Finally, the residual was redissolved prior to non-targeted metabolomics testing. Additionally, to extract the lipids, we added 20 μL of serum sample to a 1.5 mL microcentrifuge tube, followed by 120 μL of methanol solution and vortexed for 3 min. Then, 360 μLof methyl tertbutyl ether (MTBE) and 100 μL of ultra-pure water were sequentially added after oscillating for 3 min, and then the mixture was centrifuged at 13,000× *g* for 15 min. Similarly, the lipid layer was lyophilized and dissolved prior for the test. UltiMate 3000 ultra-high performance liquid chromatographic system and the Q-Exactive quadrupole -Orbitrap high resolution mass spectrometer (Thermo Fisher Scientific, Fair Lawn, NJ, USA) were used for non-targeted metabolomics analysis. For more information about metabolomics-related processes, please referred to the Supplementary Materials.

## 3. Results

### 3.1. Pan-Cancer Introduction with Respect to Differences in NMRGs

**Figure 1:**
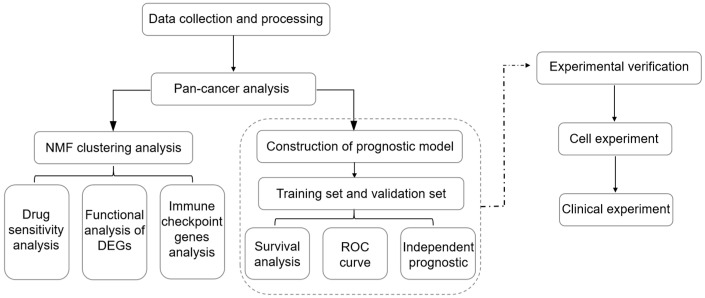
The investigation’s flow chart.

**Figure 2:**
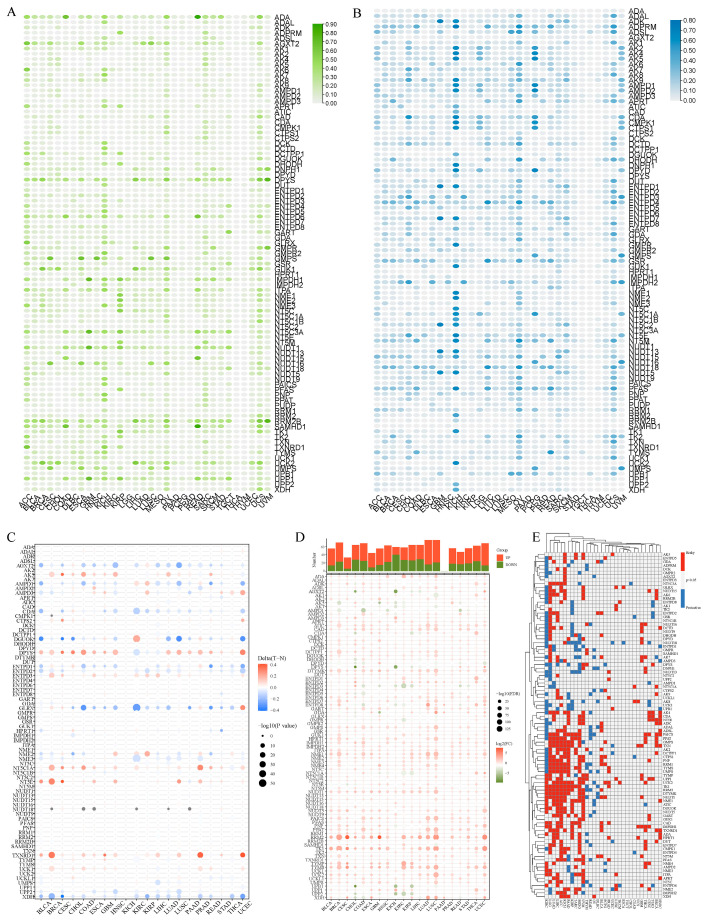
Panoramic view of nucleotide metabolism-related genes (NMRGs) in pan-cancer. (**A**,**B**) Histogram displays the frequency of copy number variation (CNV) for each NMRG in each tumor type ((**A**) amplification; (**B**) deletion). (**C**) Heatmap displays the differential methylation of NMRGs in cancers; hypermethylated and hypomethylated genes are denoted with red and blue, respectively (Wilcoxon rank-sum test). (**D**) Histogram (upper panel) and heatmap demonstrate the number of significant DEGs and the fold change and FDR of NMRGs, respectively, in each cancer. Substantially upregulated and downregulated genes are denoted with red and green, respectively. (**E**) NMRGs’ survival profiles across cancers.

A chart displaying the research steps is provided in Figure 1. TCGA availed CNV, SNV, methylation, mRNA expression profiles, and survival data for 97 NMRGs in all kinds of malignancies for the pan-cancer study. We analyzed NMRG-related SNV data to ascertain the frequency as well as the variant types in every cancer subtype. As revealed in Supplementary Figure S1A, SKCM, UCEC, LUSC, LUAD, and STAD all had substantial SNV of NMRGs. The frequency of SNV of the NMRGs was 75.17% (2703 of 3596 tumors). Missense mutations were the predominant SNP type, according to the examination of variant types. The top five mutated genes, as determined by SNV percentage analyses, were CAD, DPYD, XDH, AK9, and AMPD1, with respective mutation percentages of 8%, 8%, 8%, 7%, and 6% (Supplementary Figure S1B). Moreover, to examine the genetic aberrations of NMRGs in malignancy, the percentage of CNV was evaluated and the findings revealed that, in general, CNV occurred at remarkable frequencies in a majority of cancer types (Figure 2A,B). In addition to CNV, aberrant DNA methylation of the promoter is linked to tumorigenesis [15]. The methylation of the promoter can modulate gene expression. We observed that most NMRGs in the 20 cancer types exhibited complex methylation patterns. However, TXNRD and ENTPD3 consistently showed hypermethylation in several tumors, while NME3, UPP2, and XDH showed the opposite (Figure 2C).

Besides genetic alterations, each cancer type’s altered NMRG gene expression patterns were investigated using differential expression analysis between the malignant and nearby normal tissues. With the exception of pancreatic cancer tissues, we ascertained that most gene expression levels in cancer tissues varied in contrast with those in normal tissues. RRM2 and TK1 had remarkably increased expression levels in several cancers (Figure 2D). Afterward, utilizing univariate Cox regression of mRNA expression and OS, risky NMRGs with HR > 1 and *p*-Value < 0.05 as well as protective NMRGs with HR < 1 and *p*-Value < 0.05 were detected, as displayed in Figure 2E.

### 3.2. Identification of Differentially Expressed Prognostic NMRGs

RNA-seq data and clinical data of 49 normal samples and 373 HCC samples were retrieved from the TCGA database. A heatmap was developed with the aim of demonstrating the differentially expressed NMRGs between the normal and cancerous samples (Supplementary Figure S2A). A total of 69 out of 97 NMRGs were discovered to have differential expressions in normal and cancerous samples (Supplementary Table S2). Meanwhile, univariate Cox survival analysis was also done on NMRGs, of which 38 NMRGs were statistically significant (Supplementary Table S3). Finally, the intersection of the two sets of genes was taken to obtain 32 NMRGs for subsequent analysis (Supplementary Figure S2B).

### 3.3. NMF Clustering Identification of Molecular Typing Based on the NMRG

**Figure 3:**
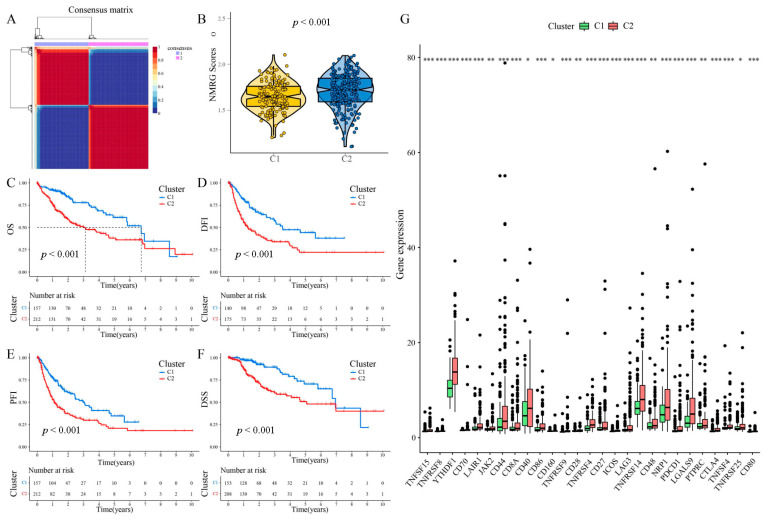
PanNMF clustering identification of two molecular subtypes with remarkably varied prognosis and expression of immune checkpoint genes. (**A**) The optimal clustering number of 2. (**B**) NMRG scores of the two subgroups are shown by violin plots. (**C**–**F**) Kaplan–Meier analyses (OS, DFI, PFI, and DSS) as regards two molecular subtypes. (**G**) Differential expression analysis of ICGs. * *p* < 0.05, ** *p* < 0.01, *** *p* < 0.001.

The NMF method selects the appropriate clustering number of two for the data, as per cophenetic, dispersion, and silhouette coefficients (Supplementary Figure S3, Figure 3A). The results of the following GSVA and KM analyses indicate that samples in C2 have higher NMRG scores and worse OS, DFI, PFI, and DSS, indicating the risky significance of NMRGs in HCC patients (Figure 3B–F). Supplementary Figure S4 shows the NMRGs that are differentially expressed in the two subgroups. Studies report adenosine block immune cell differentiation as well as maturation. It furthermore activates the expression of checkpoint molecules. We, therefore, compared the expression of ICGs between the two subtypes. Figure 3G shows all the statistically distinct ICGs, which are all expressed at higher levels in C2. In order to select appropriate administrating chemotherapeutic drugs for HCC patients, we performed chemotherapy sensitivity predictions between the two clusters. The results showed that Sorafenib, Metformin, Docetaxel, Dasatinib, Erlotinib, and Gefitinib are more suitable for C1 populations, while Gemcitabine, Doxorubicin, Cisplatin, Camptothecin, Bortezomib, and Etoposide are more suitable for C2 populations (Supplementary Figure S5).

### 3.4. Functional Analysis for the NMRG Clusters

**Figure 4:**
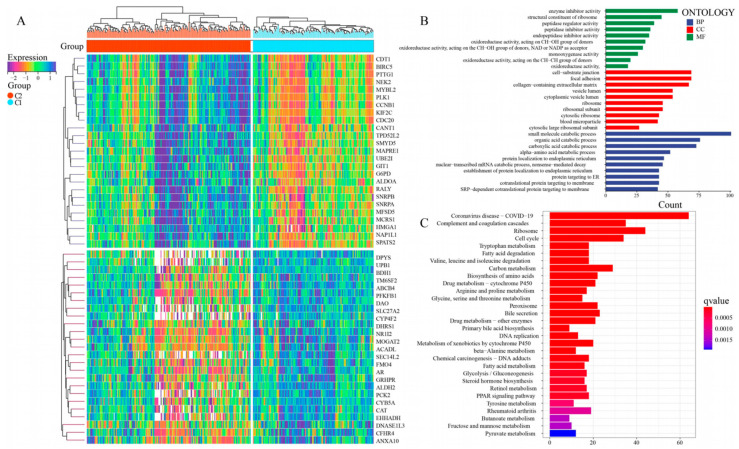
Functional analysis for the NMRG clusters. (**A**) Heatmap to display mRNA levels of DEGs in two NMRG clusters. (**B**) The analysis of GO enrichment for DEGs between two clusters. (**C**) The analysis of KEGG enrichment for DEGs between two clusters.

Then, in order to investigate probable mechanisms and biological functions at the gene level for the C1 and C2 groups, GO and KEGG pathway analyses were employed. Out of 995 DEGs that were subjected to screening (Supplementary Table S4), 356 and 639 genes were ascertained to be downregulated and upregulated in the C1 group, respectively (Figure 4A). The GO analysis affirmed that the genes were remarkably involved in the biological process of catabolic processing, inhibitor activity, and cell−substrate junction (Figure 4B). Meantime, the KEGG analysis revealed that these genes were also significantly related to various metabolic pathways, such as Tryptophan metabolism, Fatty acid degradation, Arginine and proline metabolism. (Figure 4C).

### 3.5. Determination and Verification of an NMRG-Based Prognostic Signature

**Figure 5:**
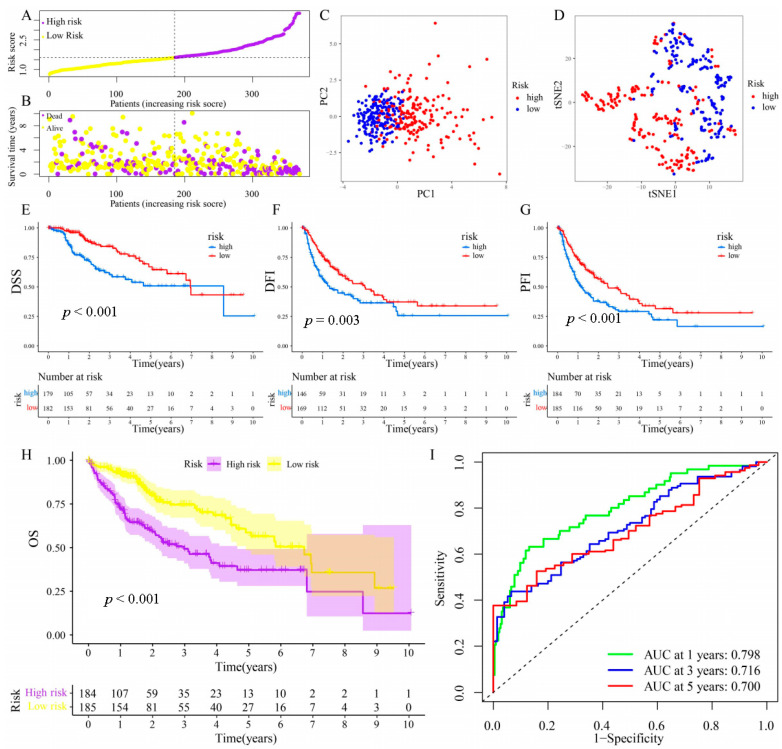
Construction of NMRG-related signature in the train cohort. (**A**) Various groups of the train cohort were created as per the median risk score. (**B**) Distributions of risk scores and the cohort’s overall survival status. (**C**) Train cohort’s PCA. (**D**) Train cohort’s t-SNE. (**E**–**H**) Kaplan–Meier analyses (OS, DFI, PFI, and DSS) of the train cohort. (**I**) The train cohort’s AUC values for ROC curves.

To examine further the prognostic value of NMRGs, NMRG-based risk scores were created to anticipate HCC patients’ survival. Upon conducting a LASSO regression and multivariate Cox analyses on the training cohort (Supplementary Figure S6), 11 genes (i.e., GMPS, UCK2, ENTPD2, PPAT, TXNRD1, RRM2, ATIC, ADSL, ADK, CDA, and DPYS) with prognostic values were uncovered from 32 NMRGs that had been previously obtained. Risk scores were subsequently determined for each HCC patient in the training cohort, and the training cohort sample was categorized into high- and low-risk subgroups based on the value of the median risk score (Figure 5A). Patients with greater risk scores had an increased likelihood of mortality, based on the risk score distributions and survival status. (Figure 5B). According to the PCA and t-SNE displayed in Figure 5C,D, patients belonging to the two risk groups may be distinguished with ease. Individuals that were in the high-risk subgroup had consistently reduced DSS, DFI, PFI, and OS values (*p* < 0.05), as shown in Figure 5E–H. Furthermore, the survival probability of the ROC curves of risk score-related AUC values were 0.798, 0.716, and 0.700 for 1, 3, and 5 years (Figure 5I), demonstrating that the risk score exerts a remarkable function in the prediction of the survival of HCC patients.

### 3.6. Predictive Efficiency of the Risk Signature Validation in the GEO Cohort

The GEO cohort (GSE14520) availed NMRG expression data on 225 HCC patients with complete survival data to confirm the replicability of the risk score in a different patient cohort. The GEO dataset was classified into high- and low-risk groups as per the median risk score of the training cohort (Supplementary Figure S7A). As displayed in Supplementary Figure S7B, the high-risk group was detected to have more death events, while the low-risk group demonstrated a remarkable probability of survival. PCA as well as t-SNE demonstrated that patients in the two risk groups were also distributed as per the two different groups (Supplementary Figure S7C,D). As demonstrated by the Kaplan–Meier curves for OS in Supplementary Figure S7E, patients in the high-risk group were discovered to exhibit a worse prognosis in contrast with the other risk group. Additionally, the high-risk group patients demonstrated a shorter survival time. A time-dependent ROC curve was examined to further determine the accuracy of the predictive risk signatures. Here, it was discovered that the AUC values of the signature in 1, 3, and 5 years were 0.611, 0.610, and 0.619, respectively (Supplementary Figure S7F).

### 3.7. Nomogram Development and Verification

**Figure 6:**
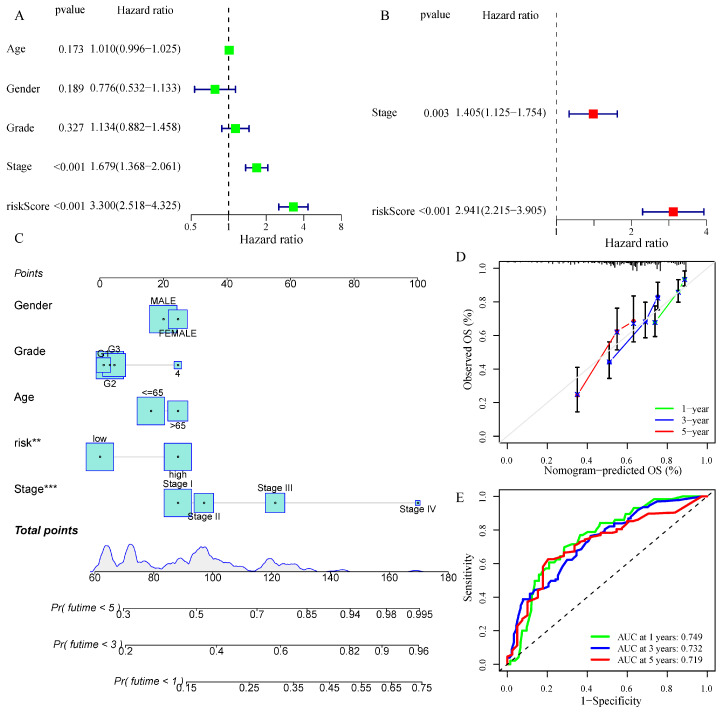
Nomogram (based on risk scores) development and verification. (**A**,**B**) Univariate and multivariate Cox regression analyses in the train cohort. (The green nodes in (**A**) indicate one-factor COX regression analysis, and the red nodes in (**B**) indicate multifactor COX regression analysis). (**C**) A nomogram of risk scores and clinical features. (The numbers in the overlapping part of (**C**) indicate the survival time (years)). (**D**) Calibration curves were utilized to validate the nomogram’s 1-year, 3-year, and 5-year predictive ability. (**E**) The AUC values of the ROC curves for improved evaluation of the nomogram’s prognostic ability. ** *p* < 0.01, *** *p* < 0.001.

To ascertain the link between immune function and the risk score, a heatmap was created. Statistically significant variations existed between the high as well as low-risk groups in the immune function of activated dendritic cells (aDCs), cytolytic activity, T cell regulation (Treg), Type I IFN Response, and Type II IFN Response in both the train and test cohorts (Supplementary Figure S8A,B). The univariate and multivariate Cox analyses evaluated the training cohort’s clinical characteristics such as age, gender, grade, stage, and risk score. The findings of the univariate Cox and multivariate Cox regression analyses revealed that the training cohort’s risk score and stage were independent prognostic predictors (Figure 6A,B). Afterward, the aforementioned factors were incorporated to generate a nomogram (Figure 6C). Furthermore, calibration curves were constructed to verify the anticipation power for the nomogram. The findings indicated an overall agreement between the nomogram’s predicted survival rates and the actual survival rates (Figure 6D). The AUC values of the nomogram in 1, 3, and 5 years for HCC were 0.749, 0.732, and 0.719, respectively (Figure 6E).

### 3.8. The Expression of Hub Gene in Different HCC Cell Lines

**Figure 7:**
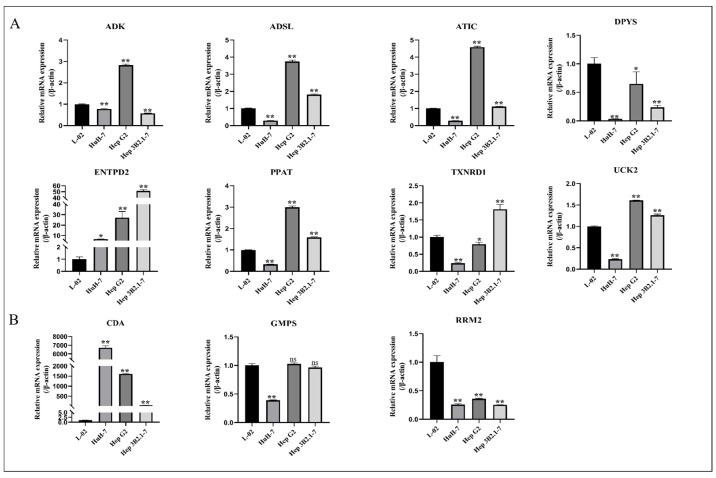
The differential expression of core genes in three hepatocellular carcinoma cell lines and normal hepatic epithelial cell lines based on RT-PCR. (**A**) Genes whose expression levels are consistent with the predicted results. (**B**) Genes whose expression levels are contrary to the predicted results. * *p* < 0.05, ** *p* < 0.01 versus L-02 group.

To validate the bioinformatics predictions, we extracted the total RNA from different human HCC cell lines (HuH7, HepG2, and Hep3B2.1–7) and human normal hepatocyte line L-02. The mRNA level of the key genes, namely ADK, ADSL, ATIC, CDA, DPYS, ENTPD2, GMPS, PPAT, RRM2, TXNRD1, and UCK2, were determined. The results showed that the expression levels of ADK, ADSL, ATIC, DPYS, ENTPD2, TXNRD1, and UCK2 in at least one tumor cell line were consistent with the predictions (Figure 7A). We found that the expression levels of CDA, GMPS, and RRM2 in HCC patients were opposite to the predicted results (Figure 7B), which is an interesting phenomenon.

### 3.9. Metabolic Profiles of Hepatocellular Carcinoma and Differential Analysis of Nucleotide Metabolites

**Figure 8:**
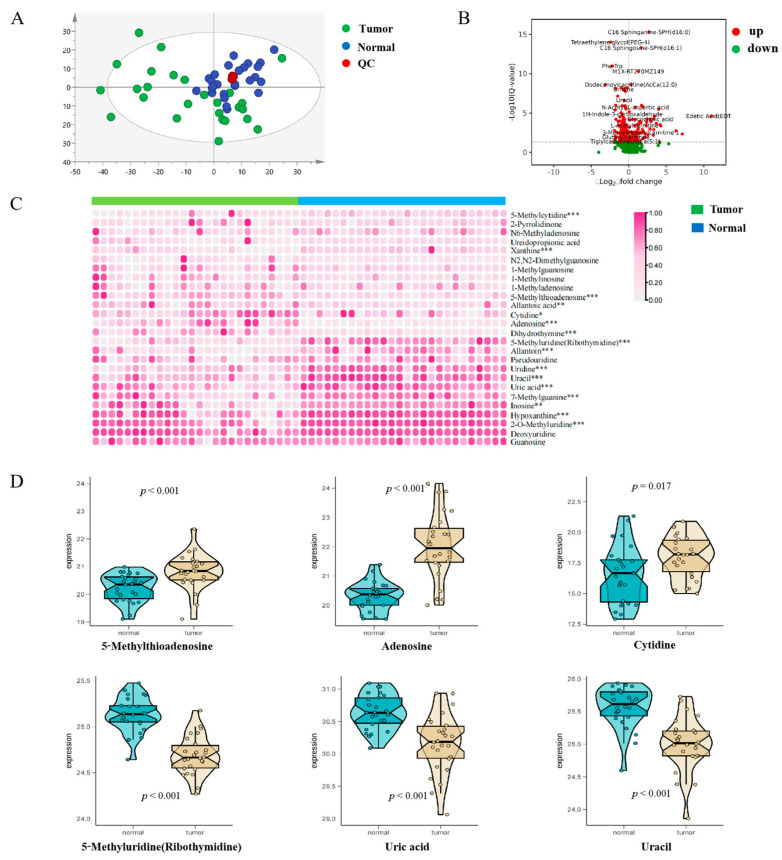
Characterization of the nucleotide metabolism landscape of hepatocellular carcinoma. (**A**,**B**) Overall metabolism of patients with hepatocellular carcinoma. (**C**,**D**) Nucleotide metabolism in patients with hepatocellular carcinoma. * *p* < 0.05, ** *p* < 0.01, *** *p* < 0.001 versus normal group.

To observe the overall metabolic profiles in patients with hepatocellular carcinoma, we performed a non-targeted metabolomics analysis. A total of 26 serum samples from HCC patients obtained from the biological sample bank of the First Affiliated Hospital of Dalian Medical University were included in this study. In addition, we matched 26 serum samples from healthy control subjects according to the sex and age of the HCC patients. Baseline information for both groups is presented in Supplementary Table S5. The results of the OPLS-DA analysis showed a significant segregation in polar metabolites and lipids for both groups (Figure 8A). Next, volcanic maps were used to perform the differences between the two groups and the mean rate of change in intensity. The results are presented in Figure 8B.

To further explore the nucleotide metabolic profile in HCC patients, we compared the levels of nucleotide-related metabolites in those two groups of the samples and the results are presented as heat maps (Figure 8C). Specifically, a total of 26 products related to nucleotide metabolism were identified, of which 16 were significantly different, as follows: adenosine, dihydrothymine, cytidine, hypoxantine, inosine, uric acid, xanthine, uridine, Uracil, Allantoin, 5-Methyluridine (Ribothymidine), 7-Methylguanine, 5-Methylcytidine, 5-MethylThioadenosine, Allantoic Acid, and 2-O-Methyluridine. We show some obviously different metabolites in Figure 8D. For the difference analysis of other metabolites, see Supplementary Figure S9.

## 4. Discussion

HCC is extremely aggressive, so it is clinically important to explore its effective prognostic indicators [16]. Recently, the traditional prognostic assessment system using clinicopathological parameters and staging has failed to meet the needs of precision medicine [17]. As sequencing technology has advanced, researchers have focused increasingly on disease molecular type and the quest for novel biomarkers to help with clinical diagnosis as well as treatment [18]. This approach not only enhances the standard prognostic assessment but also identifies a novel kind of pathogenesis. During the development of tumors, abnormal cancer metabolism takes place [19]. Recent research has demonstrated that aberrant nucleotide metabolism speeds up the progression of tumors while suppressing the tumor microenvironment’s normal immune response [7,20]. The research on the link between nucleotide metabolism and the emergence of cancer is fast progressing, despite the paucity of pertinent experiments and studies. For malignancies treatment and prevention of recurrence as well as metastasis, the intervention, change, or modulation of molecular pathways connected to aberrant nucleotide metabolism in cancerous cells has emerged as a novel strategy and idea [8]. Thus, NMRG-based risk stratification of HCC is a promising strategy for prognosis assessment and individual management.

We sum up the differences in NMRGs across numerous cancers before studying the effect of aberrant nucleotide metabolism in HCC. The differences in NMRGs more or less happened and partial NMRGs had prognostic values in various malignancies. Additionally, it was evidently shown in several tumors that NMRGs had undergone genetic mutations and alterations. NMRGs were positively correlated with MYC targets, oxidative phosphorylation, mTORC1 signaling, E2F targets, and DNA repair in a majority of types of tumors. Nevertheless, they were negatively linked to UV response DN, myogenesis, and epithelial–mesenchymal transition. MYC orchestrates proliferation, apoptosis, differentiation, and metabolism and is frequently linked to poor prognosis and survival of patients. It plays a crucial function in practically every step of the neoplastic process [21]. Ectopic MYC expression in malignancies might simultaneously promote aerobic glycolysis and/or oxidative phosphorylation to supply adequate energy and anabolic substrates that are essential for the growth of cells and cell proliferation within the tumor microenvironment [22]. In cases of proliferative deregulation and in numerous different cancer types, mTOR signaling is triggered. Numerous mTOR pathway components have been documented to be dysregulated in malignancies including breast, colon, ovarian, kidney, and head and neck cancers [23]. Recent studies in HCC and pancreatic cancer suggest that E2F expression and/or increased E2F target expression in tumors have been linked to poor prognosis [24,25,26]. Genes involved in DNA repair responses exhibit a variety of mutations and abnormal expressions in cancer cells. These changes cause genomic instability and accelerate the processes of carcinogenesis and cancer progression [27]. Aberrant nucleotide metabolism may contribute to the development of cancer by regulating the above pathways.

Then, we filtered 97 NMRGs to obtain NMRGs that were differentially expressed in both cancerous and normal tissues and had prognostic significance. Thirty-two NMRGs were found for NMF clustering and a signature building. First, 32 NMRGs are applied to divide HCC samples into two molecular clusters with significantly distinct prognoses. C2 subtype is characterized by high NMRG scores and poor prognosis (PFI, DFI, DSS, and OS), indicating the risky significance of NMRGs in HCC patients. This result is consistent with the finding that the majority of NMRGs were HCC risk genes in the pan-cancer analysis. Considering that adenosine is able to induce the expression of checkpoint molecules, we compared the differences in ICG expression between the two subtypes. We discovered that ICGs are expressed at a high level in the C2 subtype, and these differentially expressed ICGs may be intrinsic to the differential prognosis of HCC and may be potential targets for treatment. Even though there are various therapeutic choices available for HCC patients, chemotherapy remains a primary treatment modality for those with advanced HCC. Nevertheless, the efficacy of chemotherapy is yet unreliable. Therefore, it is important to find a method to accurately anticipate HCC patients’ chemotherapy responses. We then explored whether there were differences in the sensitivity of patients with two subtypes based on NMRG to commonly used chemotherapeutic agents. We found that the C1 subtype might benefit from Sorafenib, Metformin, Docetaxel, Dasatinib, Erlotinib, and Gefitinib; however, the C2 subtype might benefit from Gemcitabine, Doxorubicin, Cisplatin, Camptothecin, Bortezomib, and Etoposide. It demonstrates how NMRG-based clustering may be a huge help in accurately treating individuals with HCC.

In addition, we used the KEGG pathway enrichment analysis method to investigate the possible molecular biological mechanisms of C1 and C2 subtypes. The results showed that differential genes between subtypes were enriched in a variety of metabolic pathways, such as Tryptophan metabolism, Fatty acid degradation, Arginine and proline metabolism, Glycine, serine, and threonine metabolism, Primary bile acid biosynthesis, Fatty acid metabolism, Tyrosine metabolism, and Pyruvate metabolism among others. Dysregulation of these metabolic processes plays an important role in the development of HCC. Tryptophan catabolism has been reported to be involved in immune tolerance response and to promote response to other anticancer drugs [28]. Furthermore, altered lipid metabolism is increasingly recognized as a marker of tumor occurrence [29], and our enrichment analysis showed that fatty acid metabolic processes were indeed significantly altered. The above results give us an insight that the metabolic processes of the organism are interrelated and related to each other, while an abnormal nucleotide metabolism can lead to reprogramming situations of multiple metabolic processes, and finally jointly induce the occurrence of tumors. Therefore, focusing on the complex metabolic regulatory network may be a novel direction for predicting or treating tumors.

Additionally, to obtain a reliable signature with clinical significance, we screened 32 NMRGs by univariate Cox and LASSO regression analyses and tested the optimized candidate genes for signature development. After verification, a novel NMRG-related prognostic signature was created incorporating 11 genes (i.e., GMPS, UCK2, ENTPD2, PPAT, TXNRD1, RRM2, ATIC, ADSL, ADK, CDA, and DPYS).

Other research studies have examined these 11 genes in numerous cancer forms, some of which have also been examined in HCC. A glutamine amide is used by GMPS to generate the guanine nucleotide as part of the de novo purine biosynthesis process. Previous research has shown that GMPS was crucial to the development of ovarian cancer [30], HCC [31], myeloid [32], prostate cancer [33], etc. UCK2, which can catalyze the phosphorylation of uridine and cytidine to uridine monophosphate and cytidine monophosphate. UCK2 has been proven to enhance the migration and invasion of HCC cells [34], which was also identified to be a latent diagnostic as well as a prognostic indicator for lung cancer [35] and breast cancer [36]. ENTPD2 is regarded as a pivotal ectoenzyme engaged in extracellular ATP hydrolysis [37]. The upregulation of ENTPD2 is present in papillary thyroid carcinoma-derived cells [38], esophageal cancer cells [39], glioma cells [40], and liver cancer cells [41] in comparison to normal cells. While ENTPD2 overexpression was a poor predictor of prognosis for HCC, ENTPD2 inhibition was able to slow the progression of the tumor and improve the effectiveness and efficiency of immune checkpoint inhibitors [41]. PPAT catalyzes the initial committed step of de novo purine nucleotide biosynthesis [42,43], implying that targeting PPAT can serve as a successful cancer strategy [44]. Additionally, PPAT was discovered as a prognostic biomarker in HCC [45]. Modulation of TXNRD1 could influence the proliferation, invasion, and migration of carcinoma [46,47]. TXNRD1 is upregulated in breast cancer, head and neck cancer, and lung cancer, and its overexpression is linked to a bad prognosis [48,49]. By altering the redox balance in vitro, inactivation of TXNRD1 prevented HCC cells from proliferating and led to their apoptosis [50]. Several previous reports indicated that RRM2 functioned in the proliferation, invasion, and metastasis of malignant cells, and as a result, participated in several types of malignant tumors including HCC [51,52]. ATIC, a bifunctional protein enzyme, catalyzes the final two steps of the de novo purine biosynthesis pathway. Studies show that the overexpression of ATIC in HCC is associated with a shorter life expectancy and promotes the growth of HCC cells via controlling the AMPK-mTOR-S6 K1 signature [53]. ADSL, an essential enzyme for de novo purine biosynthesis, is thought to be a novel oncogene in prostate cancer and colorectal carcinoma [54,55]. ADK is a member of the ribokinases family and is an essential enzyme for the elimination of extracellular adenosine by phosphorylating it into 5′-adenosine monophosphate [56]. ADK can influence immune systems and aid in the development of cancer. In addition, lower ADK expression was linked to liver cancer relapse [57]. Gemcitabine became inactive as a result of the deamination of dFdC to dFdU caused by CDA [58]. According to several in vitro studies, overexpressing CDA resulted in gemcitabine resistance, whereas removing CDA restored gemcitabine sensitivity [59,60]. A zinc metalloenzyme, DPYS, which breaks down dihydropyrimidine, is expressed at a high level in tumors in contrast with the matching normal tissues [61]. According to studies, the DPYS subtype DPYSL3 was a potential biomarker for stomach cancer’s malignant nature [62].

Utilizing the signature, HCC patients may be successfully classified into the high-risk subgroup with a worse prognosis as well as the low-risk subgroup in the train, test1, test2, and test3 cohorts with a better prognosis. The areas under the ROC curves affirmed that this signature has a good predictive value. Given the possible impact of the tumor immune function on cancer therapy, we evaluated the difference in immune function between two risk subgroups of HCC. The results showed Treg and aDCs were expressed at a high level in the high-risk group, whereas the opposite was true for IFN response and cytolytic activity. To explicitly exploit the signature’s prognostic capability, the survival rate of HCC patients was quantitatively examined upon creating a nomogram based on risk score and other clinical features. ROC and calibration curves evaluated the nomogram’s predictive potential, showing high accuracy. Finally, we verified the expression of these 11 genes through basic experiments.

However, some drawbacks are related to our research. All RNA sequence data and clinical information were from public databases, such as the TCGA and GEO databases. To develop the predictive significance of our prognostic signature, substantial prospective clinical research is needed. Lastly, the feature was developed using bioinformatics research and preliminary basic experimental analysis was performed, but further genetic functional research is needed to verify our findings.

## 5. Conclusions

In this study, we successfully obtained a clinical model that can accurately predict the prognosis of HCC patients by using bioinformatics-related analysis methods. The model contains 11 NMRGs, and its expression was verified in subsequent molecular biology experiments. Finally, the nucleotide-related metabolic profile under HCC was verified in patients based on non-targeted metabolomics data. It is expected that the current investigation might provide novel perspectives for clinical management and personalized treatment of HCC patients.

## Data Availability

The data presented in this study are available in the main article, further inquiries can be directed to the corresponding authors. The data are not publicly available due to privacy.

## Supplementary Materials

The following supporting information can be downloaded at: https://www.mdpi.com/article/10.3390/metabo13111116/s1; Figure S1: Frequency of single nucleotide variations (SNVs) and various NMRG variant categories; Figure S2: Identifying NMRG-related prognostic DEGs in the TCGA dataset; Figure S3: Rank survey of NMF clustering; Figure S4: Heatmap to demonstrate mRNA levels of NMRGs in two clusters; Figure S5: The link between drug sensitivity and the NMRG clusters; Figure S6: Variable selection; Figure S7: Internal validation of NMRG-related; Figure S8: Relationship between NMRG-related signature and immune function; Figure S9: Supplement to the expression of nucleotide metabolism-related metabolites in patients with hepatocellular carcinoma; Table S1: Gene primer sequence; Table S2: The findings of DEGs between 373 HCC samples and 49 normal samples from the TCGA; Table S3: The findings of HCC samples from the TCGA database that underwent univariate Cox regression analysis; Table S4: The results of DEGs between two NMRGs clusters; Table S5: Baseline data of clinical samples.
