# Supplementary material for: A Nucleotide Metabolism-Related Gene Signature for Risk Stratification and Prognosis Prediction in Hepatocellular Carcinoma Based on an Integrated Transcriptomics and Metabolomics Approach
Source: Metabolites. 2023 Oct 30;13(11):1116. doi: 10.3390/metabo13111116 (PMC10673507; doi:10.3390/metabo13111116)
Supplement: Supplementary file 1 [file metabolites-13-01116-s001.zip › Supplementary Method.pdf]

1. Detecting parameters for polar small molecule metabolites

1.1 HPLC parameters by reversed-phase chromatography in positive mode

Metabolites were separated on an Excel 2 C18-PFP column through reversed-phase chromatography. The injection volume was set at 5 μL and the column temperature was maintained at 50°C. The mobile phase consisted of 0.1% formic acid/water (as mobile phase A) and acetonitrile (as mobile phase B), with a total flow rate of 0.4 mL/min. Initially, the mobile phase ratio was set at 98% A and 2% B, which linearly changed to 2% A and 98% B over the next 10 minutes for elution. Subsequently, a rinsing step followed by equilibration of the column took place for the next five minutes.

1.2 HPLC parameters by normal-phase chromatography in negative mode

The metabolites were separated on an Acquity HSS C18 column using reversed-phase chromatography. The injection volume was set at 5 μL and the column temperature was maintained at 50°C. The mobile phase A was ultrapure water, and the mobile phase B was 50% methanol/acetonitrile with 0.4‰ ammonium bicarbonate was added to the both mobile phases. The initial mobile phase ratio was 98% phase and 2% B, which was linearly changed to 0% A and 100% B over the next 10 min, followed by 5 min for rinsing and equilibrating. The mobile phase flow rate wat set at 0.4 mL/min.

1.3 MS parameters for polar small molecules

For different types of polar small molecules, the mass spectrometry detection parameters were similar. The anterior ionization conditions were sheath gas flow rate: 45 arb; auxiliary gas flow rate: 10 arb; ionization chamber heating temperature: 355°C; capillary temperature: 320°C; S-Lens RF level: 55%. Hydrophilic metabolites were analyzed in full-scan mode with a full-scan resolution of 70,000 FWHM, a maximum injection time of 200 ms, and a mass-to-charge ratio scan range set at 70-1000 m/z. For MS/MS spectrometry, a resolution of 17,500 FWHM was used. The tip-triggered, dynamic exclusion and isotope exclusion modes were turned on, and the precursor separation window was set at 1.0 Da. Ultra-pure nitrogen was used as the cleavage gas for collision and the overall data were collected under the above parameters.

2. Detecting parameters for lipids

2.1 HPLC parameters for the separation of lipids

Separation for lipids was performed using an Accucore C30 core-shell column. The injection volume was set at 2 μL and the column temperature was maintained at 50°C. The mobile phase A was 60% acetonitrile/water with 10 mM ammonium formate and 0.1% formic acid. The mobile phase B was 10% acetonitrile/isopropanol, containing 10 mM ammonium formate and 0.1% formic acid. The flow rate was set at 0.3 mL/min, and the mobile phase gradient was as follows (A:B): 0.01min, 90% : 10% , 5.00min, 50% : 50% ; 23.00min, 0%: 100% , 24.00min, 90% : 10% and last for 7 min.

2.2 MS spectrometry parameters for lipids

Detection of lipids was also based on the Q-Exactive combined quadrupole-orbiting ion trap mass spectrometer, with generally similar detection parameters, and some details were slightly changed as follows: the ionization source was electrospray ionization, and detection was performed in both positive and negative modes, of which the sheath gas flow rate at 45 arb, an auxiliary gas flow rate at 10 arb, the heating temperature of the ionization chamber at 355 ℃. The capillary temperature was 320 ℃, the S-Lens RF level was 55. Lipids were analyzed in full scan mode with a resolution of 70,000 FWHM, an injection time of 200 ms, and a mass-to-charge ratio scan range set at 300-2000 m/z. For MS/MS qualitative data acquisition, a resolution of 17,500 FWHM was used with a maximum injection time of 80 ms. Ultrapure nitrogen was used as the cleavage gas with a dynamic exclusion time of 8 seconds.

3. Supplementary figure legend

Supplementary Figure 1. Frequency of single nucleotide variations (SNVs) and various NMRG variant categories. (A) Mutation frequency of NMRGs. The numbers show how many samples of a certain tumour have the matching mutant gene. "0" denotes the absence of any mutation in the gene's coding region, while no number denotes the absence of any mutation in the gene at all. (B) SNV oncoplot. An oncoplot displaying the distribution of NMRG mutations and a list of SNV kinds.

Supplementary Figure 2. Identification of NMRG-related prognostic DEGs. (A) Heatmap to display the expression levels of differentially expressed NMRG between HCC and normal samples. (B) Venn diagram to find NMRG-related prognostic DEGs.

Supplementary Figure 3. Rank survey of NMF clustering.

Supplementary Figure 4. Heatmap to display mRNA levels of NMRGs in two clusters.

Supplementary Figure 5. The link between drug sensitivity and the NMRG clusters.

Supplementary Figure 6. Variable selection. (A-B) By using LASSO-Cox regression analysis, 11 genes were chosen.

Supplementary Figure 7. Internal validation of NMRG-related signature in the test cohort. (A) There was a classification of the test cohort into several categories. (B) The test cohort's survival status as well as risk score distributions. (C) PCA of test1 cohort. (D) t-SNE of test1 cohort. (E) The tests cohort’s survival curve. (F) AUC values of ROC curves in the test cohort.

Supplementary Figure 8. Relationship between NMRG-related signature and immune function in (A) train and (B) test cohort.

Supplementary Figure 9. Supplement to the expression of nucleotide metabolism-related metabolites in patients with hepatocellular carcinoma.
