# Supplementary figures and images for: A Nucleotide Metabolism-Related Gene Signature for Risk Stratification and Prognosis Prediction in Hepatocellular Carcinoma Based on an Integrated Transcriptomics and Metabolomics Approach
Source: Metabolites. 2023 Oct 30;13(11):1116. doi: 10.3390/metabo13111116 (PMC10673507; doi:10.3390/metabo13111116)

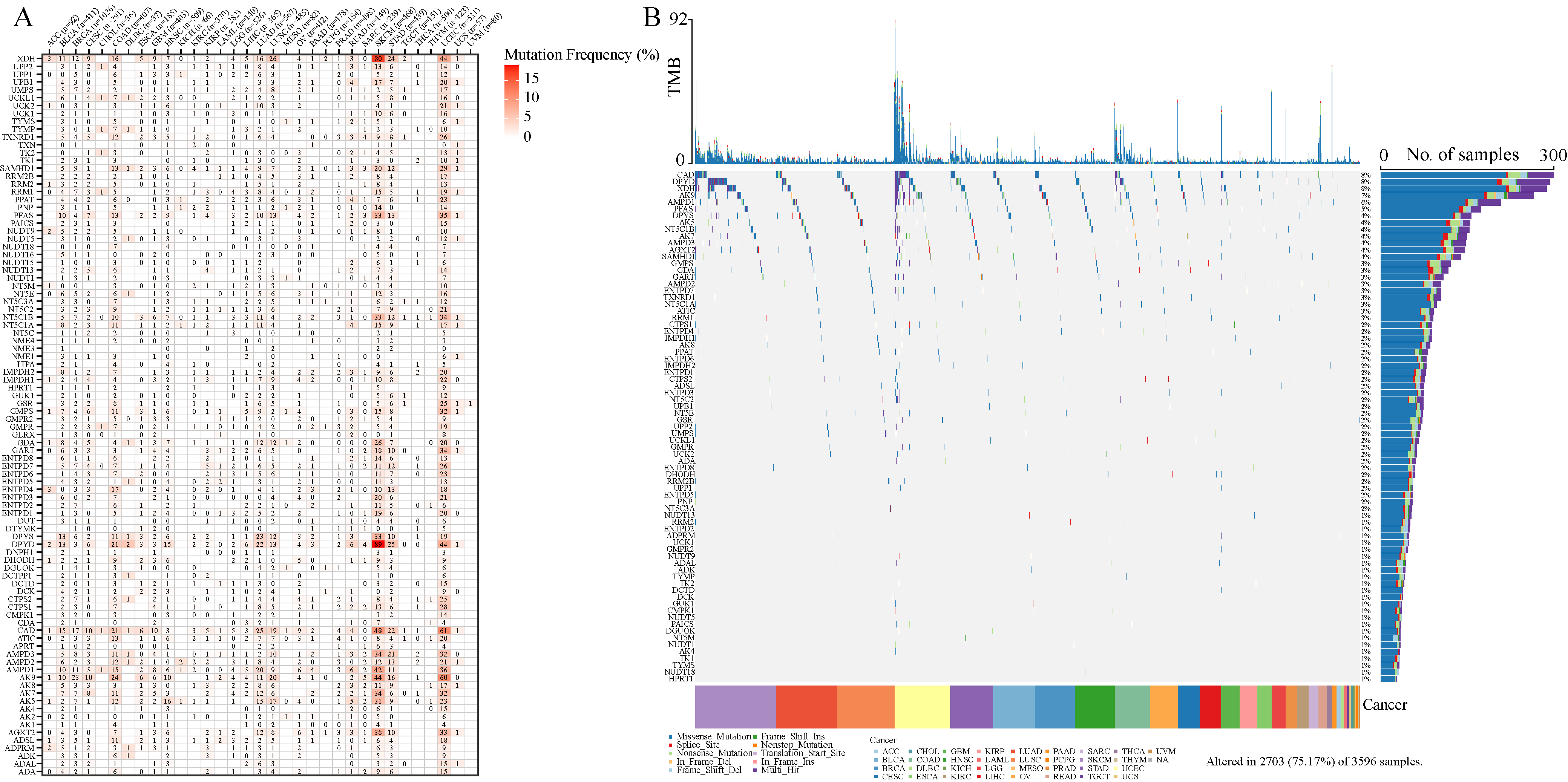

Supplement: Supplementary file 1 [file metabolites-13-01116-s001.zip › Supplementary Figure 1 Frequency of single nucleotide variations (SNVs) and various NMRG variant categories..tif]

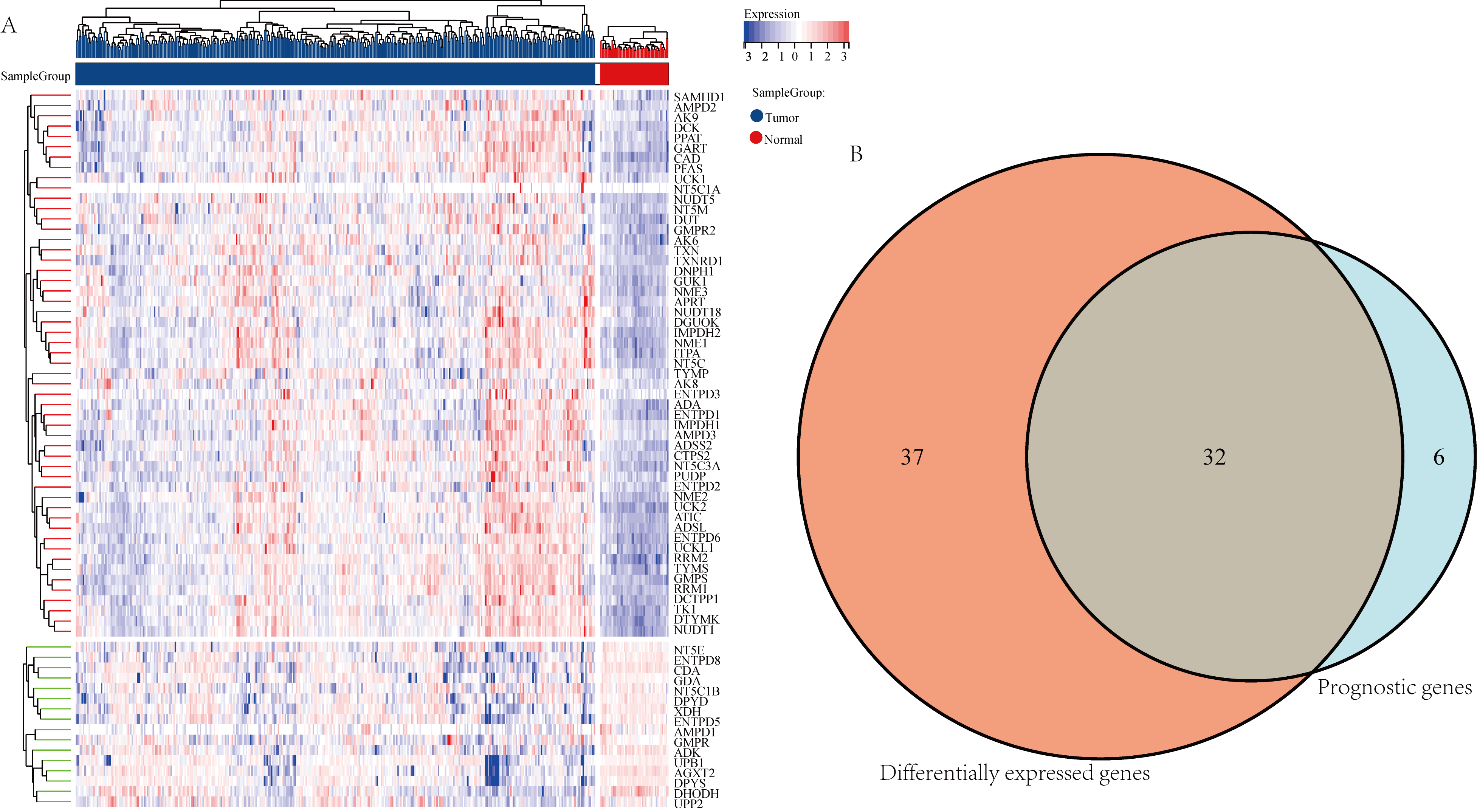

Supplement: Supplementary file 1 [file metabolites-13-01116-s001.zip › Supplementary Figure 2 Identifying NMRG-related prognostic DEGs in the TCGA dataset..tif]

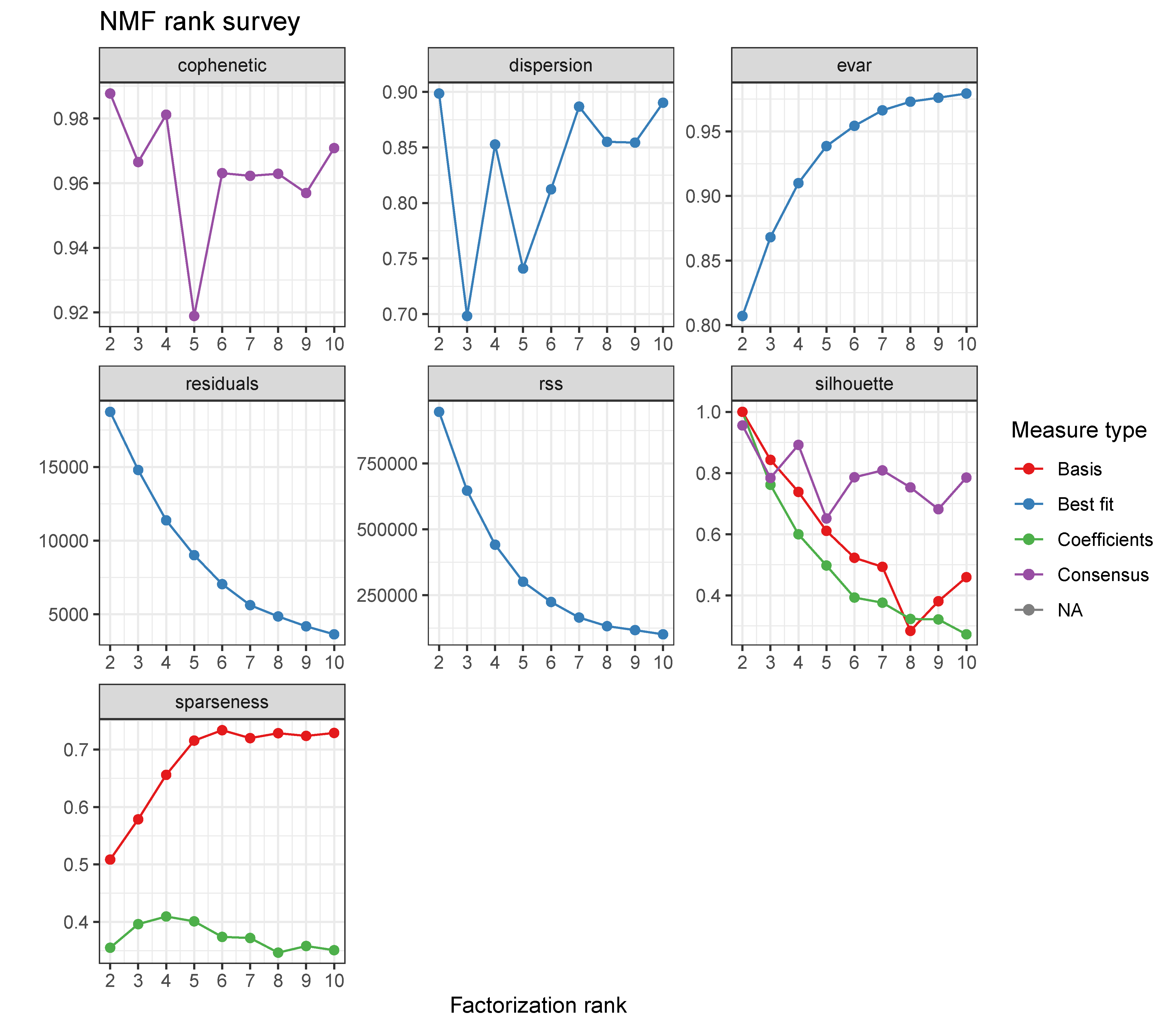

Supplement: Supplementary file 1 [file metabolites-13-01116-s001.zip › Supplementary Figure 3 Rank survey of NMF clustering..tif]

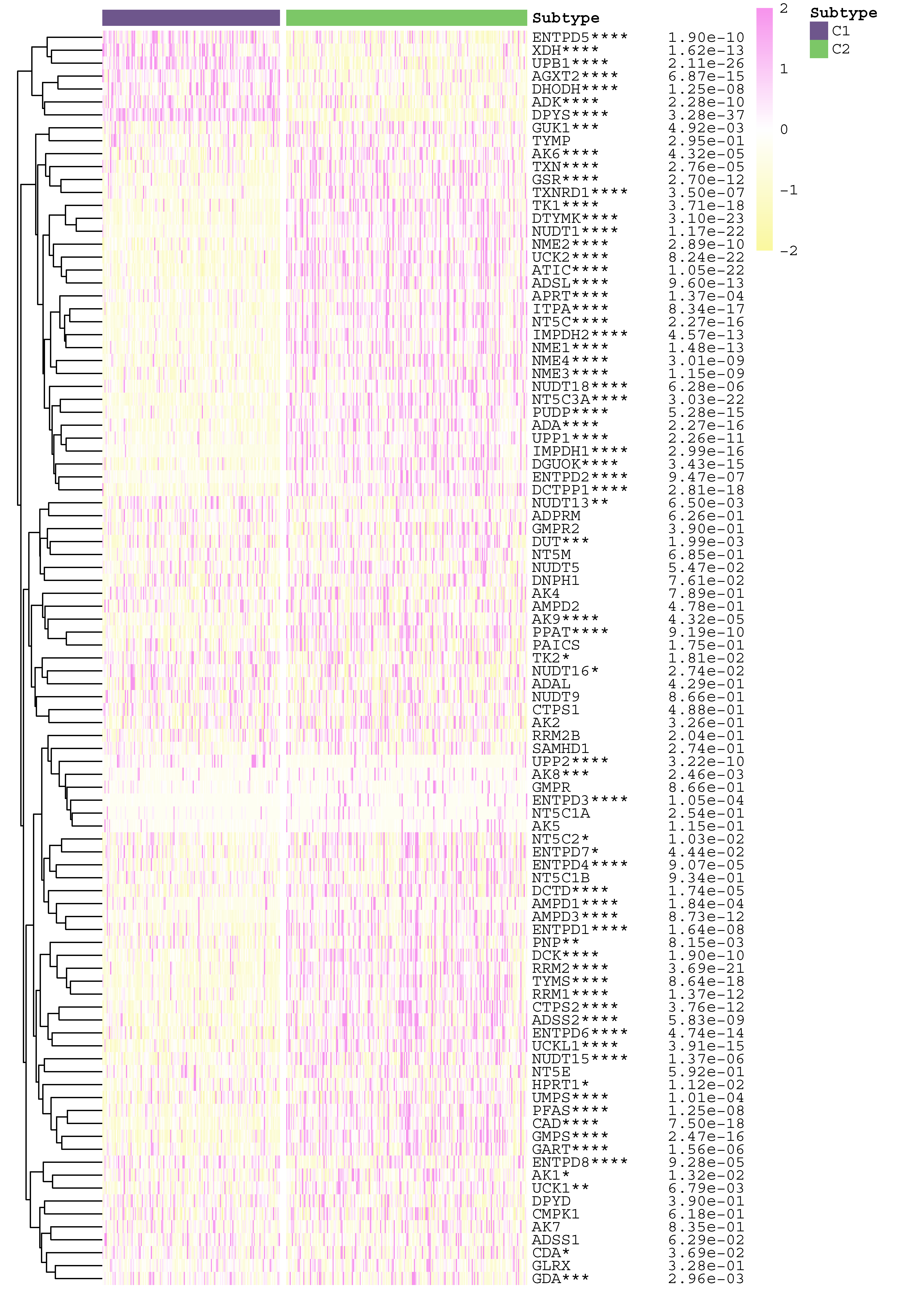

Supplement: Supplementary file 1 [file metabolites-13-01116-s001.zip › Supplementary Figure 4 Heatmap to demonstrate mRNA levels of NMRGs in two clusters..tif]

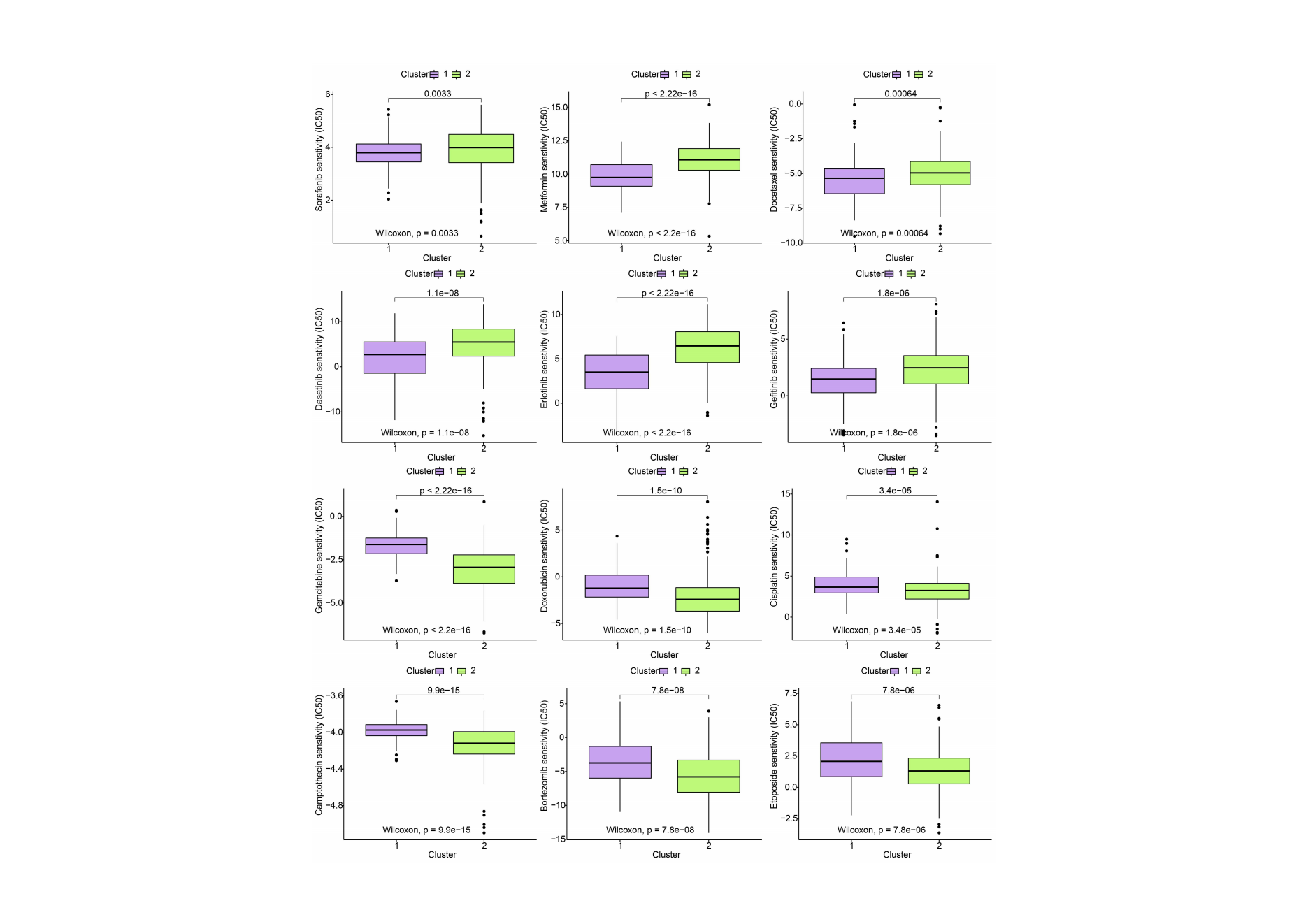

Supplement: Supplementary file 1 [file metabolites-13-01116-s001.zip › Supplementary Figure 5 The link between drug sensitivity and the NMRG clusters..tif]

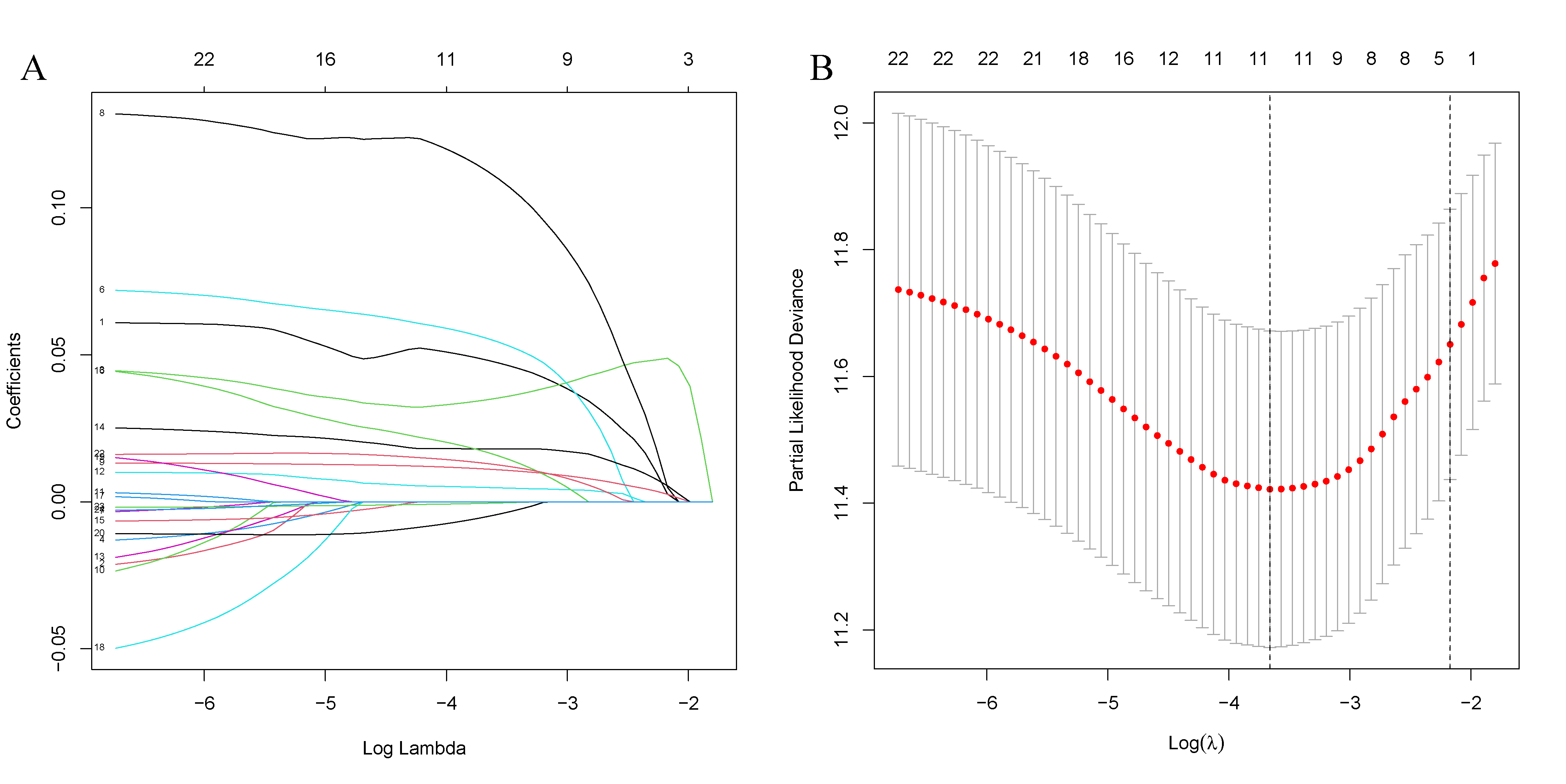

Supplement: Supplementary file 1 [file metabolites-13-01116-s001.zip › Supplementary Figure 6 Variable selection..tif]

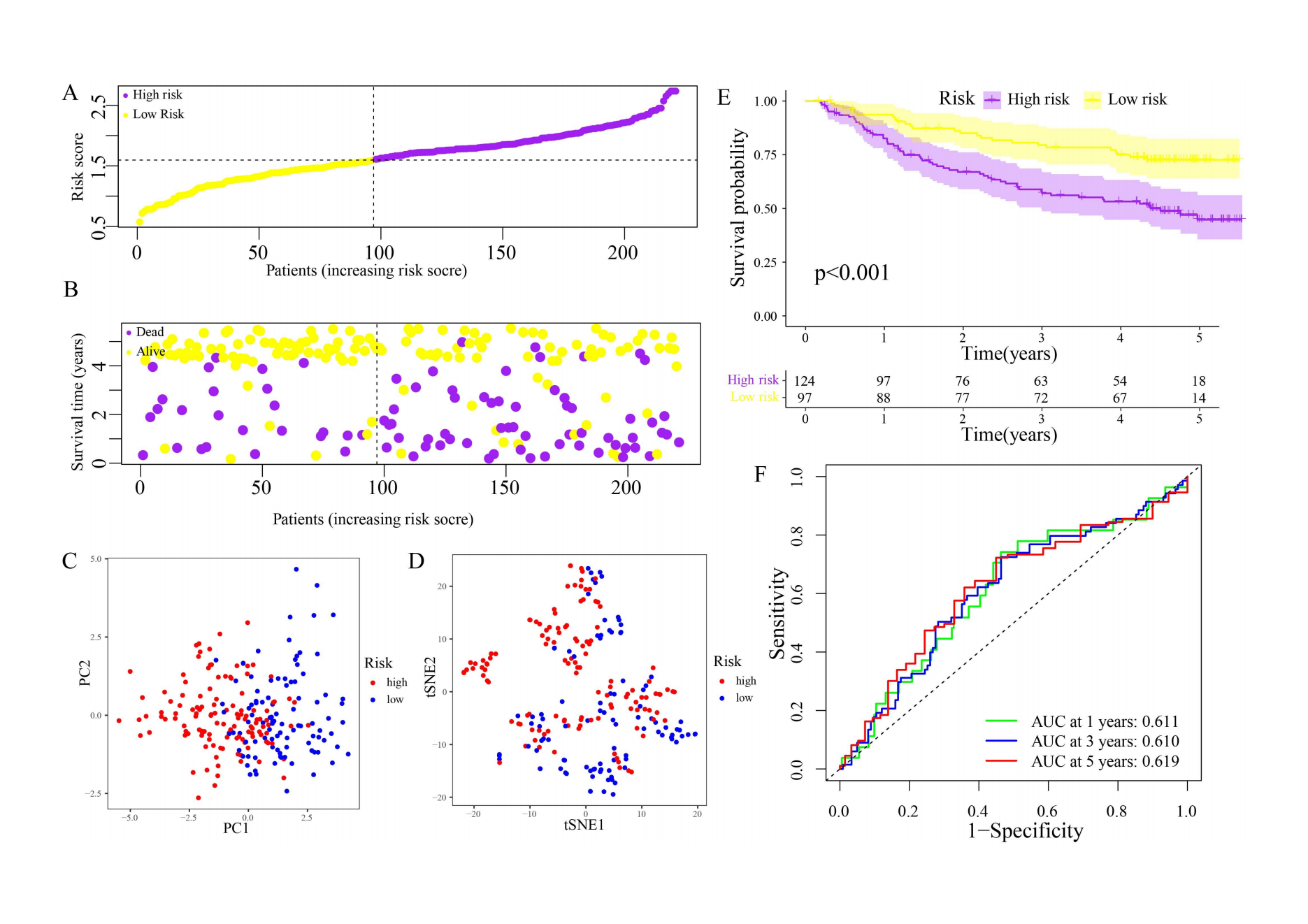

Supplement: Supplementary file 1 [file metabolites-13-01116-s001.zip › Supplementary Figure 7 Internal validation of NMRG-related signature in the test cohort..tif]

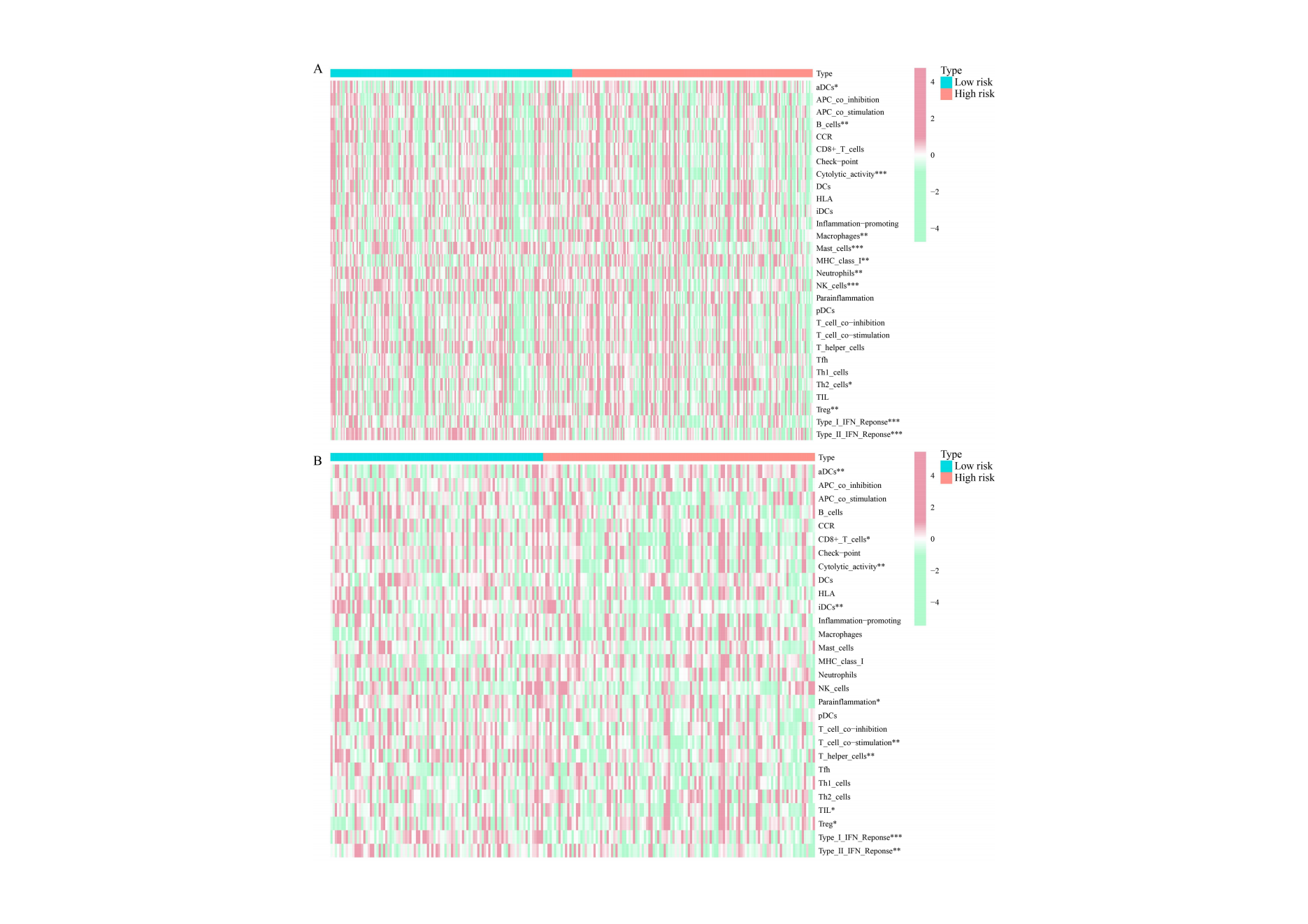

Supplement: Supplementary file 1 [file metabolites-13-01116-s001.zip › Supplementary Figure 8 Relationship between NMRG-related signature and immune function..tif]

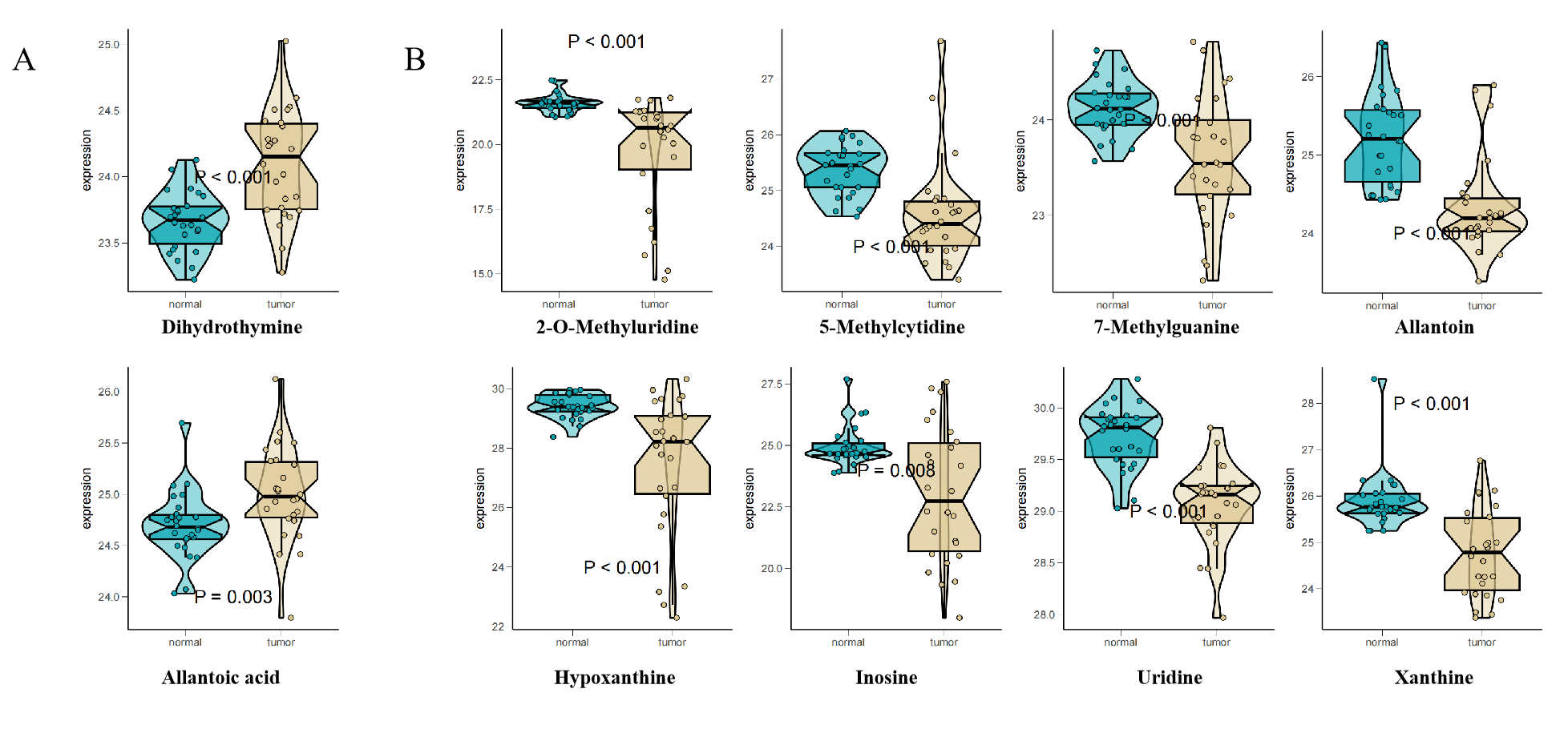

Supplement: Supplementary file 1 [file metabolites-13-01116-s001.zip › Supplementary Figure 9 Supplement to the expression of nucleotide metabolism- related metabolites in patients with hepatocellular carcinoma..tif]
